# An optimized approach for annotation of large eukaryotic genomic sequences using genetic algorithm

**DOI:** 10.1186/s12859-017-1874-7

**Published:** 2017-10-24

**Authors:** Biswanath Chowdhury, Arnav Garai, Gautam Garai

**Affiliations:** 1 Department of Biophysics, Molecular Biology and Bioinformatics, University of Calcutta, Kolkata, 700009 WB India; 2 Unit of Energy, Utilities, Communications and Services, Infosys Technologies Ltd., Bhubaneswar, 751024 Odisha India; 3 Computational Sciences Division, Saha Institute of Nuclear Physics, Kolkata, 700064 WB India

**Keywords:** Genetic algorithm, Bioinformatics, Coding region, Exon prediction, Gene identification

## Abstract

**Background:**

Detection of important functional and/or structural elements and identification of their positions in a large eukaryotic genomic sequence are an active research area. Gene is an important functional and structural unit of DNA. The computation of gene prediction is, therefore, very essential for detailed genome annotation.

**Results:**

In this paper, we propose a new gene prediction technique based on Genetic Algorithm (GA) to determine the optimal positions of exons of a gene in a chromosome or genome. The correct identification of the coding and non-coding regions is difficult and computationally demanding. The proposed genetic-based method, named Gene Prediction with Genetic Algorithm (GPGA), reduces this problem by searching only one exon at a time instead of all exons along with its introns. This representation carries a significant advantage in that it breaks the entire gene-finding problem into a number of smaller sub-problems, thereby reducing the computational complexity. We tested the performance of the GPGA with existing benchmark datasets and compared the results with well-known and relevant techniques. The comparison shows the better or comparable performance of the proposed method. We also used GPGA for annotating the human chromosome 21 (HS21) using cross-species comparisons with the mouse orthologs.

**Conclusion:**

It was noted that the GPGA predicted true genes with better accuracy than other well-known approaches.

**Electronic supplementary material:**

The online version of this article (10.1186/s12859-017-1874-7) contains supplementary material, which is available to authorized users.

## Background

Biological sequences are primarily useful computational data in molecular biology. Sequences represent symbolic descriptions of the biological macromolecules like DNA, RNA, and Proteins. A sequence provides a vital insight into the biological, functional, and/or structural data of a molecule. Therefore, the molecular information can be easily deciphered by analyzing several biological sequences. The past decade has seen a major boost in sequencing, especially after the advent of *next-generation sequencing (NGS)* technologies [1] leading to an enormous amount of nucleotide sequence data. Hence, the amount of raw, unannotated nucleotide sequence data in the databases is expanding exponentially. Therefore, the use of computational approaches to understand the functional and structural significance of these data has become vital in comparative genomics. Gene is the most important functional and structural unit of DNA. Hence, the computation of gene prediction is an essential part of the detailed genome annotation.

In an organism, DNA works as a medium to transfer information from one generation to another. A gene is a distinct stretch of DNA. It determines amino acid residues of a protein or polypeptide that is responsible for one or more biological functions of an organism. A gene undergoes transcription and translation process along with splicing to form a functional molecule or protein. Three consecutive nucleotides or a codon of a gene represents a single amino acid of a protein. A complete gene length is, therefore, always the multiplier of three. The prokaryotic gene structure consists of a long stretch of coding region and any intermediate non-coding region is absent. On the other hand, the eukaryotic gene structure is more complex. It breaks into several coding regions or exons that are separated by long stretches of non-coding regions i.e. introns. Introns are spliced out from the transcribed RNA. Furthermore, the coding region comprises only 2 – 3% of the entire genomic sequence that adds a second level of complexity in eukaryotes. As a consequence, the gene prediction in a eukaryotic genome is more challenging.

Computational gene finders are able to predict genes precisely for sequences with a single gene, but for sequences with multiple genes, the accuracy gets lowered with the increase of sequence complexity thereby resulting in false predictions. The ab-initio based method predicts the genes directly from the genomic sequences relying on two significant features: gene signals and gene content. Several well-known ab-intio programs available for gene prediction are GENSCAN [2], Genie [3], FGENESH [4], GeneId [5], GeneParser [6], GRAIL II [7], HMMgene [8], GeneMark.hmm [9], MZEF [10], AUGUSTUS [11], Morgan [12], EUI, EUI-FRAME, GI [13], and others. Among them, Genie combines information that integrates matches to homologous sequences from a protein database. However, the ab-intio based approaches normally predict a higher rate of false positive results while annotating large multi-gene genomic sequences [14]*.* In particular, ab-initio gene identifiers determine the intergenic splice sites poorly in the prediction process. Conversely, a homology-based method identifies genes by searching homologs on the databases of already established and experimentally verified coding sequences. A homology search exploits sequence alignment between genomic data and known database sequences. Currently, a large number of known protein-coding genes, cDNA, proteins, and ESTs are available in the databases. Therefore, sequence similarity based gene prediction methods are becoming useful in finding the putative genes in genomic sequences and understanding the evolutionary relationship between raw genomic data and known cDNA, proteins, or genes. A number of successful homology-based tools are FGENESH+ and FGENESH++ [4], SGP-1 [15], GenomeScan [16], GeneWise [17, 18], Procrustes [19, 20], CRASA [21], GAIA [22], SIM4 [23], Spidey [24], and others. Among them, GenomeScan, GeneWise, Procrustes, FGENESH+ (and FGENESH++), are combined tools that use the ab-initio information of a gene structure along with homology search.

Researches are still being carried out and many different techniques are getting developed to solve gene prediction problem by reducing false predictions. Acencio and Lemke [25] introduced a decision tree-based classifier and trained that with different attributes like network topological features, cellular compartments, and biological processes for finding essential genes in *S. cerevisiae*. EVidenceModeler (EVM) [26] and SCGPred [27] tools were developed as an automated eukaryotic gene structure annonator that computes weighted consensus gene structure based on multiple sources of available evidence. Genome Annotation based on Species Similarity (GASS) [28] was developed based on the shortest path model and DP to annotate a eukaryotic genome by aligning the exon sequences of the annotated similar species.

Numerical and signal representations of DNA are two other approaches where residues were converted into numerical values and ratios of signal respectively. Akhtar et al. [29] had performed symbolic-to-numeric representations of DNA and compared it with other existing techniques. Abbasi et al. [30] showed a significant improvement in accuracy of exonic region identification using a signal-processing algorithm that was based on Discrete Wavelet Transform (DWT) and cross-correlation method. Saberkari et al. [31] predicted the locations of exons in DNA strand using a Variable Length Window approach. A Digital Signal Processing (DSP) based method was used by Inbamalar and Sivakumar [32] to detect the protein-coding regions by converting DNA sequences into numeric sequences using Electron Ion Interaction Potential (EIIP). Another tool, Signalign [33] was used to convert DNA sequences into series of signal for comparative gene structure analysis.

Evolutionary algorithms like GA based techniques have also been used in solving the gene prediction problem [34, 35]. Hwang et al. [36] proposed a GA based method that maximized the partial Area Under the Curve (AUC) to predict essential genes of *S. cerevisiae* using selected features amongst 31 features. Cheng et al. [37] developed a novel machine learning based approach called feature-based weighted Naive Bayes model (FWM) that was based on Naïve Bayes classifiers, logistic regression, and genetic algorithm.

Gene identification based on expressed RNA is another growing field of research where the gene annotation is done by analyzing short RNA-seq reads derived from mRNA and mapping them to the reference genome. To get precise analysis, the sequence reads must evenly cover each transcript along its both ends. Many short read aligners are developed in the last few years like Bowtie2 [38], BWA-SW [39], and GSnap [40].

In this paper, we propose a GA based optimized gene prediction method named as *Gene Prediction with Genetic Algorithm (GPGA)*. It is a homology-based method that used in the mapping of large, unknown eukaryotic genomic sequences with the exons of known genes. The advantage of this approach is that it can be utilized in the mapping of a large genomic sequence with the help of genes present in several well-known repositories like *Ensembl* [41], *UCSC* [42] browser and others.

## Results and discussion

In the experiment, we statistically evaluated the sensitivity and specificity of GPGA at exon level on two benchmark datasets and also compared the results with other well-known and relevant techniques. Furthermore, we annotated human chromosome 21 with GPGA for a large-scale evaluation.

The proposed algorithm has been written in C and implemented on an IBM Power 6 system with 8 GB RAM per core.

### Test datasets

The performance of the GPGA method was validated on two benchmark datasets, namely, HMR195 [43], and SAG [44]. These are datasets from two different categories that possess well-annotated genomic sequences. The datasets were taken from the *GeneBench suite* [45]. A brief description of these test datasets is provided below.

The *HMR195 dataset* comprises 195 real genomic sequences of *H. sapiens, M.musculus*, *and R. norvegicus* in the sequence ratio of 103:82:10. Each sequence contains exactly one gene. The mean length of total sequences is 7096 bp. The total number of single-exon genes and multi-exon genes are 43, and 152, respectively. The total number of exons in the dataset is 948. Utilization of this dataset is shown in a wide array of researches [29, 46, 47].

*SAG dataset* is the second one tested in the experiment. It consists of a semi-artificial set of genomic sequences with 42 simulated intergenic sequences. The dataset was developed by arbitrarily embedding a typical set of 178 annotated real human genomic sequences (h178) in those 42 sequences. Each of h178 sequences codes for a single complete gene. The SAG sequences have an average length of 177,160 bp with 4.1 genes per sequence. The dataset contains total 900 exons.

### Data preprocessing (selection of homolog sets)

For experimental analysis, we compared the positions of exons found by the GPGA in the genomic sequence of a test dataset (HMR195 or SAG) with the actual positions mentioned in the corresponding annotation file provided with the datasets. For such experiment, we generated a customized dataset of homologous genes of both HMR195 and SAG. The execution of GPGA was not performed directly with the extracted exons from the genomic sequences of test datasets based on the positions mentioned in the annotation files since position comparison by this technique would have reduced the real genomic level complexity. We also did not consider RNA-seq reads in our experiment as the sequence reads are much shorter than biological transcripts and rarely span across several splice junctions [48].

**Fig. 1:**
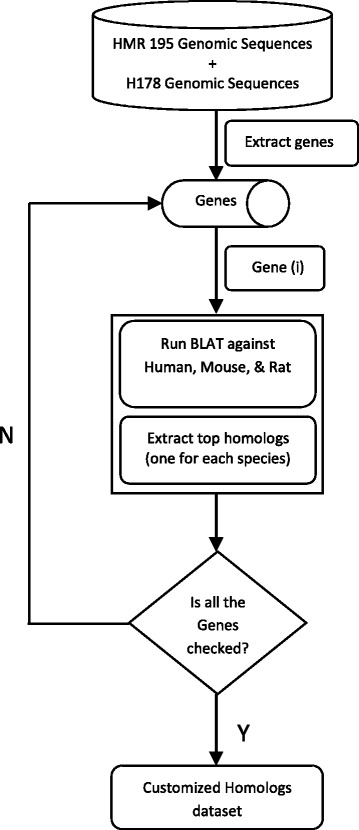
The flowchart representing the process of customized dataset construction

Three different species, namely, human, mouse, and rat were chosen for the preparation of customized homolog dataset in consideration of their phylogenetic proximity. The test datasets also contained genomic sequences drawn from these three species. To construct the customized dataset, we used Blast Like Alignment Tool (BLAT) [49] of UCSC genome browser using the default nucleotide alignment parameters. At first, all 195 and 178 genes were extracted from the genomic sequences present in the HMR and SAG datasets respectively. This was based on the positions of exons mentioned in their respective annotation files. We then searched for homologs (using BLAT) of each of the extracted genes against human, mouse, and rat genome separately using their latest assemblies (Human: hg38; mouse: mm10; and rat: rn6). From the BLAT search, we selected three highest scored homologs, each one being from each of the three considered genome assemblies. Thus, for a query gene, we got three homologs of three different species. Though we had always considered the top homologs, some of them were of poor quality in terms of similarity. Moreover, some of the homologs did not contain precise exon boundary and/or the equal number of exons of the given query presumably because the BLAT consulted newer assembled genomes compared to the genomic sequences of benchmark datasets. Despite this, all these sequences were included in the homolog sets to increase the noise in the gene data. This was done with a view to test the efficiency of the GPGA method. Multiple occurrences of same homologous sequence for different queries was eliminated from the sets to reduce redundancy. Finally, we combined the two homolog sets (one for HMR dataset and other for SAG) to generate a single customized dataset. The process flow for generating the customized dataset is shown diagrammatically in Fig. 1.

GPGA selected one exon at a time from the customized dataset and searched for its presence in both HMR and SAG datasets.

### Performance assessment

To analyze the performance, the exon positions as predicted by GPGA were compared with the actual exon positions present in the corresponding annotation file of HMR and SAG. The exon with higher alignment score is usually more accurate than the exon with low score [13]. However, in our calculation of matched results, a lower cutoff threshold was used to identify a true homolog. A minimum of 60% similarity observed between the test sequence of HMR and SAG and a sequence from the customized dataset was used as the cutoff threshold. Sometimes, it was noticed that a number of exons of a gene from the customized dataset could not individually satisfy the cutoff value. Despite this, the gene was still considered by GPGA if the combined similarity score of all exons reached to 60%. We carried out statistical analysis of experimental results to determine the performance accuracy of GPGA (see Methods for details). The results were also compared with other well-known and relevant annotation tools.

**Fig. 2:**
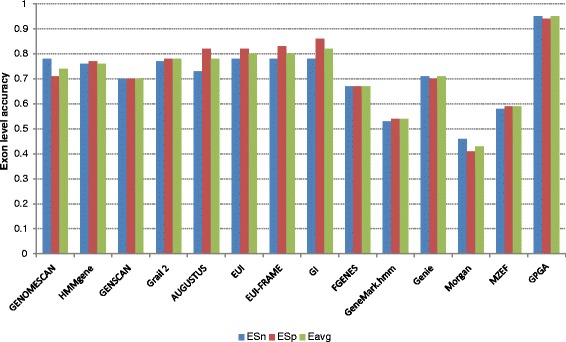
The exon level accuracy comparison of GPGA with other gene prediction tools on HMR dataset

**Fig. 3:**
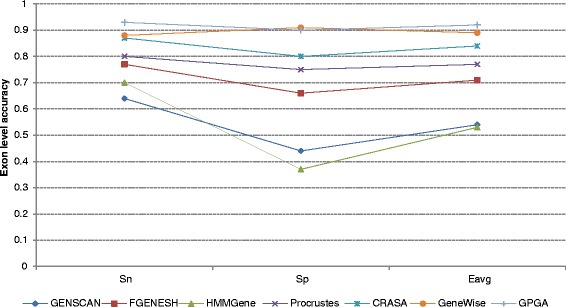
The exon level accuracy comparison of GPGA with other gene prediction tools on SAG dataset

For each test dataset, we measured *ESn* (sensitivity at the exon level), *ME* (missed exon), *ESp* (specificity at the exon level), and *WE* (wrong exon). This was done separately with human, mouse, and rat homologs from the customized dataset. The average value of *ESn* and *ESp* for each test dataset was considered for the final measurement (see Additional file 1: Statistical analysis and Table S1). Due to the presence of homologs of both the test datasets, sometimes, a number of genes from the customized dataset were not aligned with a test sequence by satisfying the cutoff similarity. In that case, the predictions based on those genes were not included in the statistical measurement of GPGA for that particular test sequence. However, we did not exclude any test genomic sequence from the measurement since the customized dataset contained at least one homolog (similarity ≥60%) that corresponds to that test sequence as described in data preprocessing. Figures 2 and 3 (Additional file 1: Tables S3 and S4) show the comparison of the GPGA results with other well-known gene prediction tools on HMR and SAG datasets, respectively. The description of each tool considered in this study was provided in Additional file 1: Table S2. In practice, it is generally difficult to compare the performance of the proposed tool with that of other gene prediction tools because most of them and their inbuilt databases are inaccessible [50]. Also, there is no facility available to incorporate any user-defined dataset. Therefore, for performance evaluation, the results of the tools were obtained from the data presented in the articles [13, 21, 43, 44, 50]. However, the selected ab-initio tools for both test datasets are trained and tested with genes of same species considered in the respective dataset. The performance of the similarity-based tools was also evaluated based on the inbuilt reference database from same or a closely related species having almost similar sequences to the test sequences. The selection of only strong homologs (above 90% similarity at the nucleotide level) yielded good accuracy for homology-based tools, whereas, selection of moderate homologs (below 70% similarity) worsened the accuracy [43, 44]. Therefore, for comparison with GPGA, we selected their best results obtained from strong homologs.

From Figs. 2 and 3, it was noticed that GPGA outperformed the other annotation tools in terms of *ESn*, *ESp*, and *Eavg*. For both the test datasets, GPGA maintained the accuracy of more than 90% for each of the three parameters. For HMR dataset, the values of *ESn*, *ESp*, and *Eavg* of GPGA were 0.95, 0.94, and 0.95, respectively. For SAG dataset, it was observed that GPGA performed similarly to GeneWise. However, the overall consistency of GPGA (*Eavg* = 0.915) was higher than GeneWise (*Eavg* = 0.89). Most of the tools were good at identifying coding nucleotides to the level of 80% or even more than 90% sensitivity and specificity (data not shown) [43, 44]. However, discovering exact exon boundary was very weak (except GeneWise) when it comes to predicting a complete gene. The exon level accuracy of GeneWise was also declined with homologs having less than 70% sequence similarity [44]. GPGA, on the other hand, was able to predict exon boundaries better than others tools even at low similarity cutoff score of 60% only. Since, all the exons are present only in the forward or plus strand the same was considered by the existing tools. However, to test the performance of GPGA, we considered both plus (Watson) and minus (Crick) strands of the test sequences.

*ME* (the proportion of missing exons and actual exons) and *WE* (the proportion of predicted wrong exons and actual predicted exons) were also included in the evaluation process for finding the accuracy of the tools. Here, GPGA also performed better than others. The results are presented in Additional file 2: Table S5 and S6. Sometimes, small exons were also missed by GPGA because of the presence of other alternative regions in the genomic sequence.

### Annotation of human chromosome 21

We also performed annotation of human chromosome 21 (HS21) to observe the performance of GPGA at the chromosome level. We selected HS21, as it is the smallest human autosome that wraps around 1-1.5% of the human genome and its structure and gene content have also been intensively studied. Therefore, it is considered as an excellent dataset to validate any gene prediction method. For cross-species comparison of HS21, we selected the phylogenetically related species, mouse. HS21 shows conserved syntenies to mouse chromosomes 10, 16, and 17 (MM-10, MM16, and MM17) [51]. Hence, we selected sequences from MM10, MM16, and MM17.

#### Data pre-processing (selection of target and reference sequences)

The main objective was to map the target sequence i.e. HS21-specific genes with their reference mouse orthologs. The entire HS21 sequence of ~47 MB (GRCh38.p4) along with its seven alternate loci (ALT_REF_LOCI_1) was obtained from the NCBI [52]. We analyzed non-repetitive parts of the HS21 sequence by aligning with well-annotated mouse CoDing Sequences (CDSs) of MM10, MM16, and MM17. The reference coding sequences were obtained from the Gencode assembly using UCSC browser [42]. GENCODE Comprehensive set is richer in alternative splicing, novel CDSs, novel exons and has higher genomic coverage than RefSeq while the GENCODE Basic set is very similar to RefSeq. Thus, we selected comprehensive Gencode VM4 published in Aug 2014.

Due to limited computing resources, we divided the entire target sequence (HS21) into multiple (total 26 numbers) divisions. Each of them consists of 16-lakh bp of HS21 except the last one. Each of these smaller divisions was run against total comprehensive sets of MM10, MM16, and MM17.

#### Results of annotation

**Fig. 4:**
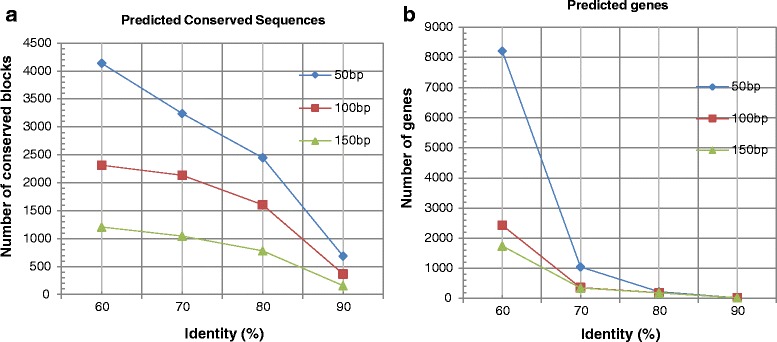
Results of Conservation identified by GPGA based on different threshold criteria; (**a**) Number of ungapped conserved blocks; (**b**) Number of genes

**Fig. 5:**
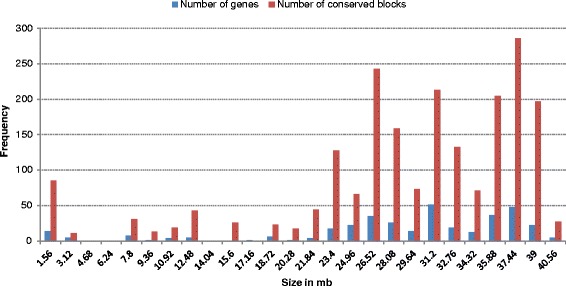
Distribution of conserved blocks and genes all along the human chromosome 21

We analyzed the results by defining different stringencies of the conserved sequences and accordingly categorized the sequences into 50, 100, and 150 bp sequence lengths. For each sequence length, we considered four types of percentage similarity, namely, 60, 70, 80, and 90. For each category of length along with its similarity, we found a large number of conserved blocks. A gene is considered to be conserved between human and mouse if all the exons of that gene satisfy the threshold criterion. For example, for a threshold criteria of 100 bp with 60% similarity, a gene with 100 bp is considered as conserved if the observed similarity is ≥60% for all mouse exons. For certain instances, it was also observed that only a few number of exons of a mouse gene individually satisfy the matching threshold criterion. We did not consider them as conserved genes but separately as conserved blocks. It is not confirmed whether these blocks are genes of HS21 or not. Figure 4a and b showed, respectively, the ungapped conserved blocks distribution and the total number of genes for different sequence lengths and similarity categories. Figure 4a supports the presence of a large number of blocks that are presumably non-genic conserved functional, regulatory, and/or structural sequences. From the figures, it was observed that for three different categories, the predicted number of conserved blocks (Fig. 4a) and genes (Fig. 4b) were decreased with the increase of the sequence length or percentage of identity. It was also noted that for a lower bp (50 bp) or low similarity value (60%) a large number of exons of the predicted genes did not follow GT-AG splicing rule. This, in turn, increased the number of false predictions. On the other hand, for a higher bp (150 bp) or high similarity value (80% or 90%), the predicted number of blocks and genes were decreased rapidly. This eventually increased the number of missed genes (details are provided in Additional file 3: Table S7). Therefore, out of all different length and similarity categories, we had finally chosen the moderate level of stringency of 100 bp with 70% identity (represented as 100-70) to increase the chance of true prediction. The importance of choosing 100-70 criterion for the identification of important elements between human and mouse was already shown in ref. [53]. The stringency of 100-70, (Table 1) yielded 2136 conserved blocks and 361 homologous genes for HS21. These 361 genes contained a total of 3150 exons out of which, 2185 exons contained canonical ‘GT-AG’ splicing junctions while the rest 604 contained non-canonical ‘GT-AG’ junctions. It was also observed that out of the 361 genes, 63 genes were overlapping genes (where both ends were not mapped by the mouse orthologs) and 149 were partial genes (having only one end matched). The calculated GC content was 51.68%, which defines the presence of GC-rich genes. Considering pseudogene based on retroposon and gene with premature stop codon we found 41 genes. The distribution of blocks and genes along the length of HS 21 is shown in Fig. 5 (see Additional file 3: Table S10). From Fig. 5, it was noted that the regions of conserved blocks and the locations of genes were close to each other and they were distributed more at the distal part (gene-rich region) of HS21.

**Table 1:** Results of GPGA for Human Chromosome 21

Stringency at 100 bp length with 70% similarity	HS21
1. Total number of conserve blocks	2136
2. Total number of genes (including partial, overlapping, and retroposon)	361
2.1. Total number of exons in all genes	3150
2.2. Number of GT-AG junctions	2185
2.3. Number of non GT-AG junctions	604
2.4. Total number of residues comprising all the genes	412,168
2.5. Total number of partial genes that have 5′ end matched	77
2.6. Total number of partial genes that have 3′ end matched	72
2.7. Total number of overlapping genes	63
2.8. Total number of retroposon (may include partial or overlap genes)	41
2.9. GC percentage	51.68

For 100-70 level, we also provided the base substitution data in Additional file 3: Tables S8, S9, and Figure. S1) that showed the higher rate of transition (substitution between two purines and between two pyrimidines) than transversion (substitution between one purine and one pyrimidine) and the higher rate of substitution at third codon position (Wobble position) than that of first and second.

To compare the GPGA with others, we considered only those genes that have either unique start or end positions. We excluded alternate transcripts having the same start and end positions. Out of the 361 genes predicted by GPGA for HS21, we found 283 genes share unique start and/or end positions. Table 2 contains the comparative results of GPGA along with other gene prediction tools. From the table, it was noticed that the GPGA predicted more genes than other gene prediction tools except GENSCAN which predicts many wrong exons.

**Table 2:** Comparative results are showing the different annotation tools along with matching genes with GPGA prediction

Gene prediction tools	Total genes	Total genes crossed 100-70 threshold level	Total genes with either unique start/end position	Number of genes matched with GPGA prediction
CCDS	339	287	238	149
AUGUSTUS	248	181	126	82
GeneID	271	122	122	85
GENSCAN	420	77	77	43
SGP Genes	271	203	203	123
GPGA Genes	361	361	283	.

The results proved the performance superiority of GPGA compared to other well-known ab-initio or homology-based approaches.

## Conclusion

GPGA is an integer based evolutionary process which simplifies the gene prediction technique. The GPGA was tested on two well-known benchmark datasets HMR195 and SAG to evaluate the performance in terms of sensitivity and specificity at the exon level. One of the datasets HMR195 consists of real genomic sequences and the other one SAG contains a semi-artificial set of genomic sequences. Such choice of datasets helps to measure the performance of an approach in a noisy environment. A major shortfall of existing homology-based methods is that the prediction accuracy may drop significantly for homologs having moderate similarity with test sequence. However, the proposed approach used in GPGA overcomes this drawback. For a moderate similarity like 60%, it was noticed that the true prediction of GPGA was better than other well-known approaches and the accuracy is more than 90%.

The limitation of GPGA is that it often fails to predict the correct position of a short length exon since the same sequence is frequently repeated in a large genomic sequence. Another shortfall of GPGA is that it performs well on an unannotated raw sequence, only when there is a good coverage of annotated information of orthologous genes. However, obtaining definite accuracy is an impossible task, because the performance of the program is very sensitive to the chosen dataset they are tested on.

In future work, we want to introduce the information of content sensors and signal sensors like GC-content value, TATA box, promoters and other compositional parameters along with the sequence homology to improve the performance of GPGA on an even more challenging dataset. We also wish to perform parallel computing for large-scale annotation without splitting the query length. In addition, we would like to observe the performance of the GPGA after introducing gaps in it.

## Methods

### Genetic algorithm

GA is one of the most commonly used evolutionary techniques for optimization. It is based on the principle of genetics and natural selection. It is an iterative method that initially starts with a set of probable solutions of a defined problem. In GA, each solution is represented by a chromosome. A set of chromosomes (also called individuals) forms a population. Each chromosome is associated with a fitness score that defines the solution quality of the problem under study. After every iteration (generation), the fittest individuals are carried on to the next generation, and this process continues until a termination criterion is satisfied. The three genetic operators: selection, crossover, and mutation help to modify a population in each generation. The conventional GA normally represents a chromosome by a binary string. Binary representation, however, can be problematic for solving some problems as it is sometimes difficult to encode a real problem with binary window. Another problem in binary coding is the increased length of the string for representing a large and complex optimization problem, which increases the computational complexity and the memory space. So, depending on the problem, other types of representation of GA apart from binary representation is necessary.

One of the most used GAs is the Real coded GA (RGA), whose significance is justified in several theoretical studies [54, 55]. In RGA, chromosomes are represented by the real numbers instead of binary numbers. Moreover, the researchers have suggested several modifications to the GA operators other than conventional one point crossover, two point crossover, bitwise flip mutation [54]. A number of such modified crossover and mutation operations have been applied in ref. [55–59] to improve the GA process for a defined problem.

Here, we have modified the conventional GA with the integer coding. The changes in crossover and mutation have also been performed for solving the problem efficiently. Such modification improves the performance of the proposed GPGA.

### Gene prediction with genetic algorithm

The objective of the proposed method (GPGA) is to map an unknown large genomic sequence with well-annotated known genes to determine any homologous relationship between the known and unknown sequence. CDSs are the important parts of eukaryotic genes and are structurally more conserved in homologous sequences. CDSs are the translated portion of a eukaryotic gene and thus consist of only exons. However, to find the small and discrete portions of CDS in a large genomic sequence is an exhaustive search procedure and requires a significant amount of computational time and memory space. We have incorporated an integer based GA (IGA) approach in GPGA to overcome such problems.

### Gene representation by GPGA

In the proposed method, the individuals of the GA population are represented by integer values. These values signify different possible positions of an exon in a large unknown genomic sequence. The searching process iteratively reaches the optimum position that defines the actual position of the exon. As a result, instead of searching the entire gene (comprising a number of exons) in an unknown genome, GPGA separately looks for each exon of the corresponding gene. Thus, the execution of GPGA is dependent on the number of exons present in a gene. This representation carries an advantage in that it breaks up the search space of the gene-finding problem to a number of smaller subspaces, thereby reducing the computational complexity. It eventually reduces the possibility to be stuck up in a local optimum.

### Population initialization

In the initialization step, an integer based initial population of size *N* is randomly generated within a lower and an upper limit. Each individual or a chromosome *P*_*i*_ *, ∀ i ∈ {1, 2,…, N}* is an integer value that represents a probable location of an exon (*E*) in the query genomic sequence (*Q*). The lower and the upper limits define the lowest and the highest probable exon’s position in *Q*. The lower limit (*l*) defines the starting position of *Q* i.e., 1. The upper limit (*u*) is the difference between the length of *Q* and the exon (*E*) length, i.e., if the length of *Q* is *q* and the length of *E* is *e*, the upper limit *u* is (*q* – *e*).

### Fitness function

The fitness score of a chromosome represents the alignment score. The alignment finds the presence of a conserved region (exon) in the query sequence. In the score calculation, we have considered that an identical match gets +1, and a mismatch gets a 0. Thus, the score is computed by the following fitness function,

1 $$ F=\varSigma {w}_i,\forall i\in \left( 1, 2,\dots, n\right) $$

where *w*_*i*_ defines a local alignment score and *n* is the total number of local alignments. *w*_*i*_ > 0, if any locally matched portion is found, otherwise, *w*_*i*_ = 0.

**Fig. 6:**
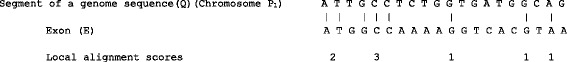
Fitness score calculation in GPGA

Therefore, the fitness value (*F*) of a chromosome denotes the summation of all local alignment scores. Now, let the chromosome be *P*_*1*_. The fitness score calculation of *P*_*1*_ is shown in Fig. 6.

Figure 6 shows five local alignment scores for *P*_*1*_. According to the Eq. 1, the fitness score of *P*_*1*_ is *F (P*_*1*_ *)* = 2 + 3 + 1 + 1 + 1 = 8.

### Genetic operators

Three genetic operators namely, *selection*, *crossover,* and *mutation* play an important role towards the convergence of the problem. These operators also maintain a balance between the exploration and exploitation of the search space.

#### Selection operator

In GPGA, we have considered tournament selection technique with tournament size 3 as a selection operator. In this approach three individuals are chosen randomly from the population pool *P*_*i*_ *, ∀ i ∈ {1, 2,…, N}* and are entered into the tournament. Based on the fitness value, the fittest individual among three will be selected to take part in the crossover operation. This process is continued along with crossover and mutation until an entirely new population *P’*_*j*_ *, ∀ j ∈ {1, 2,…, N}* is generated.

#### Crossover operator

In the GPGA, we have considered a modified crossover operation named as *Adaptive Position Prediction (APP)* crossover. APP crossover is a self-controlled-crossover operation that adaptively modifies *l* and *u* depending on the fitness scores of parents. Let us consider two parents (say, *P*_*a*_ and *P*_*b*_) are randomly selected from the population pool. Now, let, the fitness (alignment) score of *P*_*a*_ and *P*_*b*_ be *P*_*a*_^*obj*^ and *P*_*b*_^*obj*^, respectively. By this operation, two offsprings (say, *P’*_*a*_ and *P’*_*b*_) are generated from the selected parents. To generate offsprings, APP crossover can narrow down the *l* and *u* if the *P*_*a*_^*obj*^ and *P*_*b*_^*obj*^ are high. However, the *maximum fitness score* of a parent will never exceed *e* (the length of the exon). If the score is *e*, then it is considered that the optimal exon region is found and the exon *(E)* is entirely overlapped. On the other hand, if the score is either close to *e*, then it is considered as the suboptimal exon region and a part of the exon *(E)* is overlapped. Then the APP crossover narrows down the range of limits *l* and *u* close to the parents to search for offsprings. The default cutoff score for a suboptimal exon region is selected as 50% of the *maximum fitness score,* i.e.*, e*/2. On the other hand, if *P*_*a*_^*obj*^ and *P*_*b*_^*obj*^ are less than *e*/2, then *P’*_*a*_ and *P’*_*b*_ are randomly produced by choosing random positions from the unmodified *l* and *u*.

Thus, the crossover operation helps to predict the correct exon position by adaptively narrowing down the difference between *l* and *u*. This adaptive nature helps in fine-tuning of the operator for converging to the optimal position.

The APP crossover operation is represented algorithmically in the following way.
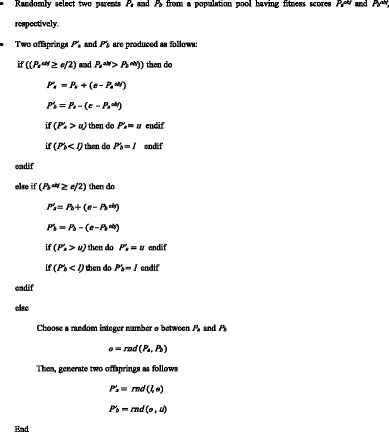


#### Mutation operator

The mutation operation is performed similarly to the APP crossover. It is also named as Adaptive Position Prediction (APP) mutation. It mutates the offspring generated from the crossover operation to another possible offspring to maintain the diversity in the population for faster searching for the optimal position of the given exon (*E*). Let, the fitness score of an offspring *P’*_*a*_ be *P’*_*a*_^*obj*^. If *P’*_*a*_^*obj*^ ≥ *e*/2, then the modified offspring (*P″*_*a*_) is generated from the narrowed down, new lower limit (*l*_*m*_) and new upper limit (*u*_*m*_).

However, if *P’*_*a*_^*obj*^ < *e*/2, then *P″*_*a*_ is generated randomly from the unmodified *l* and *u*.

The algorithmic steps of the APP mutation operation are given below.
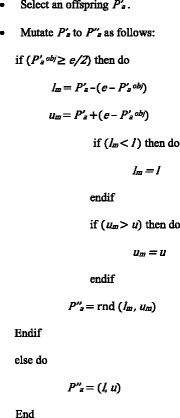


### Termination

The process is terminated when the maximum number of iterations *(generations)*, *G*_*max*_ is reached. However, to reduce the computation time without compromising the accuracy level, another termination criterion based on the fitness score of the best individual is set. If the score of the best solution remains unchanged for 200 consecutive generations, then the process is stopped.

Now, the proposed GPGA has represented algorithmically in the following way.

1. Read the unknown genomic sequence *(Q)* and the reference exon sequence (known) *(E)* which is to be mapped*.*
2. Initialize the population size *N*, AAP crossover probability *(P*_*cross*_), AAP mutation probability *(P*_*mut*_) and *G* = 1
3. Generate an initial population *P*_*i*_ *, ∀ i ϵ {1, 2,…,N}* of *N* individuals(chromosomes). Where each chromosome represents a probable starting position of *E* in *Q*.
4. Evaluate the potential of each individual *P*_*i*_ *, ∀ i ϵ {1, 2,…,N}* in terms of fitness score based on the objective function *F* (discussed in Fitness Function).
5. Select individuals from the pool of *N* individuals using the tournament selection with *tournament size* 3 and pick up two best individuals *P*_*a*_ and *P*_*b*_ based on fitness value.
6. Perform the AAP crossover operation (discussed in *Crossover operator*) with *P*_*cross*_ between the selected individuals *P*_*a*_ and *P*_*b*_ and mutate them (discussed in *Mutation operator*) with mutation probability, *P*_*mut*_.
7. Each pair of the individual (*P*_*a*_ and *P*_*b*_) generates two children *P’*_*a*_ and *P’*_*b*_ .
8. Repeat steps 5 – 7 until a new pool of individuals *P’*_*i*_ *, ∀ i ϵ {1, 2,…,N}* is formed and *G = G + 1*.
9. Stop the process if the termination criterion is satisfied (discussed in Termination). Otherwise, go to step 4.

### GPGA parameters

In the proposed method, we considered the values of *N* = 200 and *G*_*max*_ = 3000. Since the computational time increases with *G*_*max*_ value, we set the termination criterion based on the convergence of the best fitness score (see Termination). This approach always prevents the unwanted computation of GPGA up to *G*_*max*_. The optimum value of *N* was set to 200 as it produced the best results in the experiment. For GPGA, we allowed crossover and mutation operations to perform in every iteration to converge faster to an optimal solution. As a result, we set up *P*_*cross*_ = 1, and *P*_*mut*_ = 1. This eventually relieves the user to choose specific values for *P*_*mut*_ and *P*_*cross*_. Thus, the user with less or no prior knowledge of the GA can run GPGA very easily without concerning about the optimal values of *p*_*cross*_ and *p*_*mut*_.

### Evaluation of prediction accuracy

Gene prediction accuracy of GPGA was computed at the level of exons. We followed the standard measures of sensitivity (*ESn*, and *ME*) and specificity (*ESp*, and *WE*) for evaluating the performance accuracy as described previously [60], and are formulated below.

2 $$ \mathrm{Sensitivity}(ESn)=\frac{\mathrm{Number}\  \mathrm{of}\  \mathrm{correctly}\  \mathrm{predicted}\kern0.5em \mathrm{exons}\ \left(\mathrm{CE}\right)}{\mathrm{Number}\  \mathrm{of}\  \mathrm{actual}\  \mathrm{exons}}, ME=\frac{\mathrm{Number}\  \mathrm{of}\  \mathrm{Missing}\  \mathrm{exons}}{\mathrm{Number}\  \mathrm{of}\  \mathrm{Actual}\kern0.5em \mathrm{exons}} $$

3 $$ \mathrm{Specificity}(ESp)=\frac{\mathrm{Number}\  \mathrm{of}\  \mathrm{correctly}\  \mathrm{predicted}\  \mathrm{exons}\ \left(\mathrm{CE}\right)}{\mathrm{Number}\  \mathrm{of}\  \mathrm{predicted}\  \mathrm{exons}},\kern0.36em WE=\frac{\mathrm{Number}\  \mathrm{of}\  \mathrm{Wrong}\  \mathrm{exons}}{\mathrm{Number}\  \mathrm{of}\  \mathrm{Predicted}\kern0.5em \mathrm{exons}} $$

4 $$ \mathrm{Average}(Eavg.)=\left( ESn+ ESp\right)/2 $$

The predicted exon is regarded as correct only if its both sides’ boundaries are predicted correctly.

## Additional files

Additional file 1: Statistical analysis and Table **S1**-**S4**. **Table S1**. Performance analysis of GPGA on different benchmark datasets. Considered statistical parameters are Missed exon ratio (ME), Wrong Exons ratio (WE), Sensitivity (ESn), Specificity (ESp), and Average (EAvg). **Table S2**. A summary of the description of each tool considered in this study for comparison with the proposed method (GPGA). Name of the tools are mentioned alphabetically. **Table S3**. Comparative analysis of different gene prediction tools on the HMR195 dataset. Numbers of sequences are carefully selected for which the tools were defined so that the tools analyzed the sequences effectively. **Table S4**. Comparative analysis of different gene prediction tools on the SAG dataset.(PDF 136 kb)

Additional file 2: **Table S5.** Comparative analysis of missed exons and wrong exons on HMR195 datasets. **Table S6**. comparative analysis of missed exons and wrong exons on SAG datasets. (XLS 30 kb)

Additional file 3: Accuracy of GPGA in Human Chromosome 21 annotation; Table S7-S10; Figure S1 (a) and (b). **Table S7**. Total number of conserve blocks, exons, genes (along with overlapping/partial genes), and pseudogenes of HS21 predicted by GPGA for different stringency criteria. **Table S8**. The analysis of residue substitution position in a triplet codon. **Table S9**. percentage of residue substitution (between A and T, A and G, A and C, T and G, T and C, G and C) along with its positional preference in a codon for the final stringency criterion (100-70). **Table S10**. Distribution of conserved blocks and genes along the length of HS21. **Figure S1 (a)**. schematic representation of positional biasness for substitution in a triplet codon; **(b)**. Schematic representation of the rate of base substitution between A and T; A and G; A and C; T and G; T and C; G and C. (PDF 202 kb)

## Abbreviations

A: Adenosine
APP: Adaptive Position Prediction
BLAT: Blast Like Alignment Tool
bp: Base pair
C: Cytidine
cDNA: complementary DNA
CDS: CoDing Sequence
DNA: DeoxyriboNucleic Acid
DP: Dynamic Programing
Eavg: average at Exon level
ESn: Sensitivity at Exon level
ESp: Specificity at Exon level
EST: Expressed Sequence Tag
G: Guanosine
GA: Genetic Algorithm
GPGA: Gene Prediction with Genetic Algorithm
HMM: Hidden Markov Models
HMR: Human: Mouse: Rat
HS21: Homo Sapience 21
ME: Missed Exon
MM 10,16,17: *Mus musculus* 10, 16, 17
NGS: Next-Generation Sequencing
RefSeq: Reference Sequence
RNA: RiboNucleic Acid
RNA-seq: RNA sequenceing
SAG: Semi Artificial Genome
T: Thymidine
WE: Wrong Exon
