# Supplementary material for: An optimized approach for annotation of large eukaryotic genomic sequences using genetic algorithm
Source: BMC Bioinformatics. 2017 Oct 24;18:460. doi: 10.1186/s12859-017-1874-7 (PMC5655831; doi:10.1186/s12859-017-1874-7)
Supplement: Supplementary file 1 — Statistical analysis and Table S1-S4. Table S1. Performance analysis of GPGA on different benchmark datasets. Considered statistical parameters are Missed exon ratio (ME), Wrong Exons ratio (WE), Sensitivity (ESn), Specificity (ESp), and Average (EAvg). Table S2. A summary of the description of each tool considered in this study for comparison with the proposed method (GPGA). Name of the tools are mentioned alphabetically. Table S3. Comparative analysis of different gene prediction tools on the HMR195 dataset. Numbers of sequences are carefully selected for which the tools were defined so that the tools analyzed the sequences effectively. Table S4. Comparative analysis of different gene prediction tools on the SAG dataset.(PDF 136 kb) [file 12859_2017_1874_MOESM1_ESM.pdf]

Statistical Analysis:

SAG (The average value of each parameter was calculated separately for human, mouse, and rat homolog set):

ESn = (0.9366+0.9300+0.9179)/3 = 0.9282

ESp = (0.9155+0.8892+0.8868)/3 = 0.8972

EAvg = (0.93+0.90)/2 = 0.92

ME = (0.0156+0.0356+0.0433)/3 = 0.0315

WE = (0.0367+0.0764+0.0744)/3 = 0.0625

HMR195 (The average value of each parameter was calculated separately for human, mouse, and rat homolog set):

ESn = (0.9515+0.9620+0.9314)/3 = 0.9483

ESp = (0.9426+0.9502+0.9226)/3 = 0.9385

EAvg = (0.95+0.94)/2 = 0.95

ME = (0.0095+0.0074+0.0169)/3 = 0.0113

WE = (0.0184+0.0192+0.0257)/3 = 0.0211

Table S1: Performance analysis of GPGA on different benchmark datasets. Considered statistical parameters are Missed exon ratio (ME), Wrong Exons ratio (WE), Sensitivity (ESn), Specificity (ESp), and Average (EAvg).

**DATASET**		**EXON LEVEL ACCURACY**				
**Dataset name**	**Total no. of sequences**	**ME**	**WE**	**ESn**	**ESp**	**Eavg**
**HMR195 dataset**	195	0.011	0.021	0.95	0.94	0.95
**SAG dataset**	43	0.032	0.063	0.93	0.90	0.92

Table S2: A summary of the description of each tool considered in this study for comparison with the proposed method (GPGA). Name of the tools are mentioned alphabetically.

Program	Type	Description	Organisms	Algorithm used	Homology
**AUGUSTUS**	Ab-initio	It is able to predict multiple splice variants. It evaluates based on the hints (evidences) to potentially protein-coding regions by means of a Generalized Hidden Markov Model (GHMM) that takes intrinsic information. It can integrate extrinsic information (information	Human, fly, Arabidopsis	GHMM	

		from RNA-Seq, ESTs, and proteins). In our case, we used no extrinsic information.			
**CRASA**	Homology-based	It enables direct alignment of cDNA sequences to the genome.	Human	BLASTX	cDNA
**EUI**	Combinatorial Ab-initio	It considers the exons predicted by Genscan and HMMgene and labeled them as exons if exon probability scores greater than the defined threshold value. Even when the exon probability scores are less than threshold, the program labeled them as true predictions only when both Genscan and HMMgene predict them as exon.	-	Genscan and HMMgene	
**EUI_frame**	Combinatorial Ab-initio	It applies EUI method to the Genscan and HMMgene predictions to maintain the reading frame consistency. If a gene predicted by Genscan overlaps with a gene predicted by HMMgene, this program chooses the one with higher gene probability to impose reading frame.	-	Genscan and HMMgene	
**FGENES v. 1.6 (FGENESH v. 2.6)**	Ab-initio	It uses *linear discriminant analysis* (LDA) to determine a signal of an exon. LDA is a mathematical technique that first combines the data of multiple experiments, and then uses a linear function to discriminate two classes of events: exon and intron. FGENES uses Dynamic Programming (DP) to determine the best combination of predicted exons into a gene model. (FGENESH is a variant of FGENES that uses HMM instead of LDA.)	Human	LDA, DP (HMM, DP)	
**GeneMark.hmm v. 2.2**	Ab-initio	The optimal gene candidates selected by the GHMM and DP	Human, mouse, and others	GHMM and DP	
**GeneWise v. 2.1.16b**	Homology-based	It is based on global alignment at the level of translated ORF/protein.	Human	DP (dynamite)	Protein
**Genie v. 2.1**	Combined	It uses generalized HMM with arbitrary length distributions associated with some states of the model. The probabilities for gene features are estimated by using DP	Human, mouse, and others	GHMM and DP	Protein

		that combine information from multiple content and signal sensors, including sensors that integrate matches to homologous sequences from a database.			
**GenomeScan**	Combined	It incorporates protein homology information by running GENSCAN and then compares the results to known proteins using BLASTP or BLASTX.	Vertebrates	Genscan method, BLASTP or BLASTX	Protein
**Genscan v. 1.0**	Ab-initio	It uses a GHMM probabilistic model with fifth-order Markov chain for gene structure evaluation. It also has the ability to optimize the predictions of either partial genes or multiple genes separated by intergenic DNA.	Human	GHMM	
**GI**	Combinatorial Ab-initio	It considers only those exons that belong to regions predicted as genes by both programs: Genscan and HMMgene.	-	Genscan and HMMgene	
**Grail II**	Ab-initio	It uses variable length windows tailored to each potential exon candidate, defined as an open reading frame bounded by a pair of start/donor, acceptor/donor or acceptor/stop sites.	Human, mouse, Drosophila	Neural Network (NN)	
**HMMgene v. 1.1d**	Combined	It is based on HMM, and is trained using a criterion, called conditional maximum likelihood, which maximizes the probability of correct prediction. If a sequence's subregion is identified as coding region based on the observed similarity with ESTs, cDNAs, or proteins in a database, these regions are locked as coding regions and then they are submitted to HMMgene.	Human	HMM	Protein, cDNA, or EST
**MORGAN v. from April 1998**	Ab-initio	It used a decision tree classifier that classifies subsequences into different classes: start codons, donor sites, and acceptor sites and these are brought together in a frame-sensitive DP algorithm that finds the optimal segmentation of a DNA sequence into coding and noncoding regions (exons and	Vertebrates	Decision trees, DP, and Markov chains.	

		introns).			
**MZEF v. from April 1998**	Ab-initio	MZEF uses a *quadratic discriminant function* (QDA) to distinguish between two classes: coding and noncoding.	Human	QDA	
**PROCRUSTES v. 4.0**	Homology-based	It is based on protein/protein alignments scored with PAM 120.	Vertebrates	DP	Protein

Table S3: Comparative analysis of different gene prediction tools on the HMR195 dataset. Numbers of sequences are carefully selected for which the tools were defined so that the tools analyzed the sequences effectively.

**PROGRAM**	**Number of Sequences**	**EXON LEVEL ACCURACY**				
		**ME**	**WE**	**ESn**	**ESp**	**Eavg**
**GENOMESCAN**	195	0.05	0.14	0.78	0.71	0.74
**HMMgene**	195	0.12	0.07	0.76	0.77	0.76
**GENSCAN**	195	0.08	0.09	0.70	0.70	0.70
**Grail 2**	50	0.07	0.06	0.77	0.78	0.78
**AUGUSTUS**	195	0.17	0.08	0.73	0.82	0.78
**EUI**	195	0.10	0.04	0.78	0.82	0.80
**EUI-FRAME**	195	0.11	0.03	0.78	0.83	0.80
**GI**	195	0.19	0.03	0.78	0.86	0.82
**FGENES**	195	0.12	0.09	0.67	0.67	0.67
**GeneMark.hmm**	195	0.13	0.11	0.53	0.54	0.54
**Genie**	195	0.19	0.11	0.71	0.70	0.71
**Morgan**	127	0.20	0.28	0.46	0.41	0.43
**MZEF**	119	0.32	0.23	0.58	0.59	0.59
**Proposed method (GPGA)**	195	0.011	0.021	0.95	0.94	0.95

Table S4: Comparative analysis of different gene prediction tools on the SAG dataset.

PROGRAM	Number of Sequences	EXON LEVEL ACCURACY				
		ME	WE	ESn	ESp	Eavg
**GENSCAN**	42	0.14	0.41	0.64	0.44	0.54
**Fgenesh**	42	0.09	0.23	0.77	0.66	0.71
**HMMGene**	42	0.15	0.55	0.70	0.37	0.53
**Procrustes**	42	0.10	0.16	0.80	0.75	0.77
**CRASA**	42	.	.	0.87	0.80	0.84
**GeneWise**	42	0.06	0.02	0.88	0.91	0.89
**Proposed method (GPGA)**	42	0.032	0.063	0.93	0.90	0.92
