# Supplementary material for: An optimized approach for annotation of large eukaryotic genomic sequences using genetic algorithm
Source: BMC Bioinformatics. 2017 Oct 24;18:460. doi: 10.1186/s12859-017-1874-7 (PMC5655831; doi:10.1186/s12859-017-1874-7)
Supplement: Supplementary file 3 — Accuracy of GPGA in Human Chromosome 21 annotation; Table S7-S10; Figure S1 (a) and (b). Table S7. Total number of conserve blocks, exons, genes (along with overlapping/partial genes), and pseudogenes of HS21 predicted by GPGA for different stringency criteria. Table S8. The analysis of residue substitution position in a triplet codon. Table S9. percentage of residue substitution (between A and T, A and G, A and C, T and G, T and C, G and C) along with its positional preference in a codon for the final stringency criterion (100-70). Table S10. Distribution of conserved blocks and genes along the length of HS21. Figure S1 (a). schematic representation of positional biasness for substitution in a triplet codon; (b). Schematic representation of the rate of base substitution between A and T; A and G; A and C; T and G; T and C; G and C. (PDF 202 kb) [file 12859_2017_1874_MOESM3_ESM.pdf]

Accuracy of GPGA in Human Chromosome 21 annotation

We have performed a comparative analysis of the entire human chromosome 21 with the syntenic regions of the mouse genome i.e. chromosomes 10, 16 and 17. Since, Gencode consortium has proven to contain more rich and correct genes than Refseq, thus the reference coding sequences are obtained from UCSC browser from the latest Gencode assembly i.e. comprehensive Gencode VM4 published in Sep 2013.

The results are as follows:

Table S7: Total number of conserve blocks, exons, genes (along with overlapping/partial genes), and pseudogenes of HS21 predicted by GPGA for different stringency criteria.

Set	Conserved Blocks	Total residues	Exons			Genes	Partial genes			Pseudogene	GC %
			Total	GT-AG	Non GT-AG		5' end	3' end	both ends	Retrotransposon	
**50-60**	4136	1511300	32384	2882	21284	8218	839	1885	4970	90	45.11
**50-70**	3239	450522	4557	2186	1322	1049	97	158	626	42	50.74
**50-80**	2449	204862	1641	1199	217	225	32	45	47	15	50.97
**50-90**	687	18170	129	91	17	21	4	1	2	1	50.49
**100-60**	2315	1104632	18833	2843	13561	2429	388	515	1251	82	46.49
**100-70**	**2136**	**412168**	**3150**	**2185**	**604**	**361**	**77**	**72**	**63**	**41**	**51.68**
**100-80**	1607	202901	1580	1199	187	194	31	44	18	14	50.95
**100-90**	368	18170	129	91	17	21	4	1	2	1	50.49
**150-60**	1209	1021111	16518	2829	11944	1745	161	396	1007	78	46.58
**150-70**	1047	410779	3129	2180	599	350	74	69	62	37	51.69
**150-80**	782	201806	1561	1194	182	185	30	41	17	12	50.96
**150-90**	161	17924	123	89	15	19	3	1	2	1	50.57

Table S8: The analysis of residue substitution position in a triplet codon

Set	Total Mutated residue	Residue substitution						Codon Position substitution		
		A-T	A-G	A-C	T-G	T-C	G-C	First	Second	Third
**50-60**	474684	80873	102053	73575	67706	87272	63205	148011	141563	185110
**50-70**	92523	10297	25530	12084	9591	23209	11812	23721	19956	48846
**50-80**	30007	2403	8957	3383	2514	9416	3334	6262	4322	19423
**50-90**	1465	110	448	148	103	534	122	209	129	1127
**100-60**	333616	54354	69894	51144	46858	66699	44667	99733	95222	138661
**100-70**	**82036**	**8275**	**22251**	**10789**	**8212**	**21732**	**10777**	**20012**	**16682**	**45342**
**100-80**	29669	2385	8662	3374	2506	9411	3331	6204	4223	19242
**100-90**	1465	110	448	148	103	534	122	209	129	1127
**150-60**	301308	48758	63235	45713	41521	62129	39952	88911	84464	127933
**150-70**	81809	8266	22182	10754	8180	21664	10763	19957	16644	45208
**150-80**	29523	2384	8616	3350	2488	9365	3320	6177	4207	19139
**150-90**	1452	110	444	147	102	530	119	207	129	1116

Table S9: percentage of residue substitution (between A and T, A and G, A and C, T and G, T and C, G and C) along with its positional preference in a codon for the final stringency criterion (100-70)

Set	Total Mutated residue	Residue substitution						Codon Position substitution		
		A-T (%)	A-G (%)	A-C (%)	T-G (%)	T-C (%)	G-C (%)	First (%)	Second (%)	Third (%)
100-70	82036	10.09	27.12	13.15	10.01	26.49	13.14	24.39	20.34	55.27

Figure S1 (a): schematic representation of positional biasness for substitution in a triplet codon; (b): Schematic representation of the rate of base substitution between A and T; A and G; A and C; T and G; T and C; G and C.

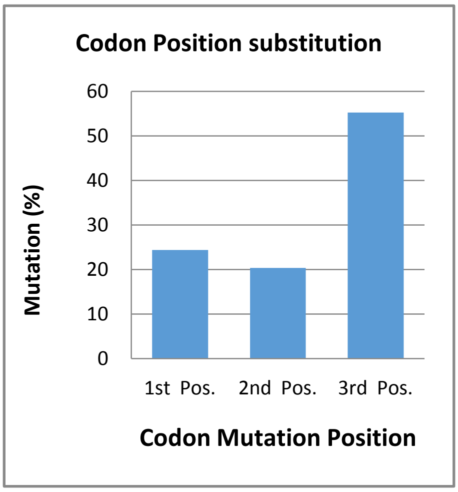

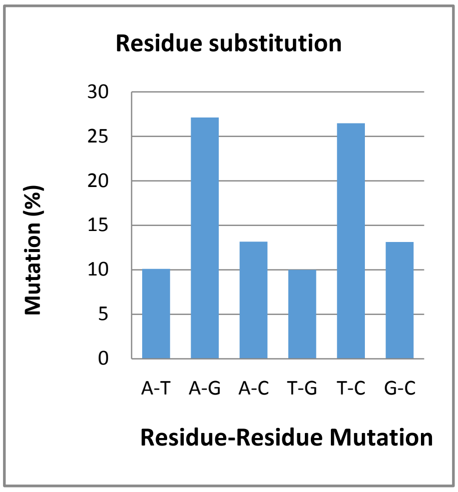

Table S10: Distribution of conserved blocks and genes along the length of HS21

Set	Sl. No.	Base pair size (bp)in lakh	File size (mb)	Number of genes	Number of conserved blocks	Number of exons
Sequence (Genes or Conserved Blocks) >= 100bp and identity >= 70% (100_70).	1	16	1.56	14	85	140
	2	32	3.12	5	11	7
	3	48	4.68	0	0	0
	4	64	6.24	0	0	0
	5	8	7.8	7	31	39
	6	96	9.36	1	13	24
	7	112	10.92	4	19	9
	8	128	12.48	5	43	23
	9	144	14.04	0	0	0
	10	160	15.6	0	26	0
	11	176	17.16	1	0	3
	12	192	18.72	6	23	31
	13	208	20.28	1	17	8
	14	224	21.84	4	44	20
	15	240	23.4	17	128	133
	16	256	24.96	22	66	83
	17	272	26.52	35	243	328
	18	288	28.08	26	159	287
	19	304	29.64	14	73	116
	20	320	31.2	51	213	410

	21	336	32.76	19	133	195
	22	352	34.32	12	71	106
	23	368	35.88	36	205	298
	24	384	37.44	48	286	413
	25	400	39	22	197	386
	26	416	40.56	5	27	44
	Alt. loci			6	23	47
